# Seed Hydropriming and Smoke Water Significantly Improve Low-Temperature Germination of *Lupinus angustifolius* L.

**DOI:** 10.3390/ijms19040992

**Published:** 2018-03-26

**Authors:** Agnieszka Płażek, Franciszek Dubert, Przemysław Kopeć, Michał Dziurka, Agnieszka Kalandyk, Jakub Pastuszak, Bogdan Wolko

**Affiliations:** 1 Department of Plant Physiology, University of Agriculture, Podłużna 3, 30-239 Kraków, Poland; kubapaaa@gmail.com; 2 Polish Academy of Sciences, Institute of Plant Physiology, Niezapominajek 21, 30-239 Kraków, Poland; dubert@ifr-pan.edu.pl (F.D.); przemyslawkopec@gmail.com (P.K.); m.dziurka@ifr-pan.edu.pl (M.D.); a.kalandyk@interia.pl (A.K.); 3 Polish Academy of Sciences, Institute of Plant Genetics, Strzeszyńska 34, 60-479 Poznań, Poland; bwol@igr.poznan.pl

**Keywords:** abscisic acid, amylase, cold, dehydrogenases, gibberellins, membrane permeability, narrow leaf lupine, seed germination, smoke water

## Abstract

Seed imbibition under cold temperature is dangerous when dry seeds have relatively low water content. The aim of this study was to investigate germination of 20 lines/cultivars of narrow-leaf lupine at 7 °C (cold) and 13 °C (control) under the influence of smoke water and following seed hydropriming for 3 h at 20 °C. The efficacy of individual treatments was examined with regard to seed protection during low-temperature germination. Based on seed germination, vigour at cold was evaluated four days after sowing by means of hypocotyl length, the studied lines/cultivars were divided into three groups with low, high and very high germination rates. Germination vigour correlated with cell membrane permeability, dehydrogenase activity and abscisic acid (ABA) content and was analysed in the seeds one day after sowing. Gibberellin content did not correlate with germination vigour. The seeds of weakly germinating lines/cultivars had the highest cell permeability and ABA content as well as the lowest amylolytic activity at both studied temperatures. Additionally, the vigour of weakly germinating seeds at 7 °C correlated with dehydrogenase activity. Three-hour hydropriming was the most effective for seed germination under cold due to reduced cell membrane permeability and ABA level. Stimulating effects of smoke water on germination under cold could be explained by enhanced dehydrogenase activity.

## 1. Introduction

Over the last few years, several national programs founded by the Polish Ministry of Agriculture and Rural Development (e.g., “Polish protein” project) or the National Centre of Research Development (e.g., “Segenmas”) have been implemented in Poland to reactivate cultivation of leguminous plants as valuable sources of protein. The main focus of these programs has been the cultivation of lupines, i.a. *Lupinus angustifolius* (narrow-leaf lupine), that are popular due to their reliable maturation as summer-grown crops, reasonable disease resistance, good yields and easy adaptation to various environmental conditions. Narrow-leaf lupine favourably affects soil structure due to its long root system, ability to absorb nitrogen due to symbiotic *Bradyrhizobium* bacteria, and high protein content [1]. 

One of the most important environmental determinants of plant growth is temperature, especially during seed germination. Seed imbibition, which occurs rapidly, is a key stage of the germination process. Water is absorbed by hydrophilic cell-wall components, cytoplasm and storage compounds, especially proteins and carbohydrates. Temperature has a significant impact on the pace of the imbibition. The higher the temperature, the faster the seeds imbibe. Imbibition in cold water is not only slow but also dangerous to cell membranes non-adapted to such thermal conditions: they cannot withstand water pressure and may rupture. Water influx into cells of desiccated seeds temporary perturbs the membranes, which may lead to changes in their selectivity. A result of this process is an immediate, rapid leakage of ions and solutions of low molecular weight metabolites from the cells [2]. 

Hexagonal arrangement of membrane phospholipids in dry seeds makes the plasma membranes exceedingly permeable. Imbibition at low temperatures, when water content of dry seeds is relatively low (below 10% of dry matter), is especially dangerous [3]. Seed storage proteins, such as those of soybean, are particularly sensitive during imbibition in the cold [4]. Damage caused by rapid water influx into cells may be limited if the seeds are osmoconditioned and humidified at a higher temperature [5]. Another way to minimize damage is a gradual acclimation to cold, which initiates rebuilding of cell membranes, mainly by promoting changes in fatty acid composition. Greater incorporation of unsaturated fatty acids into cell membranes increases membrane flexibility and therefore, reduces their fragility. Membrane reorganization at low temperatures requires at least a few days [6]. Typically, however, seeds are sown immediately into cool soil without acclimation. This is the case of narrow-leaf lupine seeds in Poland that are sown in March, when soil temperature does not exceed 5 °C.

The germination process is controlled by many external environmental factors (light, temperature and moisture) as well as by internal growth regulators, such as gibberellins (GAs) and abscisic acid (ABA). Germination depends on the ratio of GAs to ABA. Gibberellins break dormancy and induce expression of genes encoding enzymes such endo-β-1,3-glucanase, β-1,4-mannan endohydrolase, and, especially in grains, α-amylase [2,7]. These enzymes hydrolyse endosperm and promote inhibitory effects of ABA on embryo growth potential [2]. Abscisic acid takes part in seed maturation, seed dormancy and germination. Seed maturation involves inhibition of the cell cycle, a decrease in cell moisture, an increase in ABA levels and finally, an established dormancy. Primary dormancy results from ABA activity during seed development. This hormone inhibits seed germination by affecting the cell cycle. Therefore, seeds deficient in ABA germinate better. The cell cycle is related to residual G_1_ kinase which is activated in the absence of ABA [8]. Additionally, ABA inhibits seed germination through delaying the radicle expansion and weakening of the endosperm as well as enhancing expression of transcription factors that may adversely affect seed germination [9]. 

Respiration is one of the first initiated metabolic processes during imbibition of germinating seeds. Its individual stages are activated gradually: glycolysis is initiated during the first few hours, and it is followed by the Krebs cycle. Both processes provide energy needed for de novo synthesis of many compounds. Dehydrogenases play an indirect role in oxidative phosphorylation, transferring hydrogen protons from appropriate substrates to NAD^+^ coenzyme (nicotynamide adenine dinucleotide, oxidized form), thereby reducing it to reduced NADH + H^+^. The latter transfers protons to the oxidative chain.

Karrikins (KAR) are a chemically defined family of plant growth regulators discovered in smoke from burning plant material [10,11]. Most studies researching positive effects of smoke on seed germination were performed on native species of Australia, South Africa and California, USA. Smoke water (SW) may break seed dormancy in many species, such as the members of *Fabaceae*, *Convolvulaceae* and *Rhamnaceae* families [12]. Studies by Rokich et al. [13] and Van Staden et al. [14] demonstrated that cold smoke water had a similar effect on seed germination as an active compound in the smoke known as butenolide, while karrikins are derivatives of butenolide [15]. According to Soós et al. [16] SW contains many active components that exert positive or negative effects on germination and seedling vigour of numerous crop plants. Zhou et al. [11] showed some differences in seed germination evoked by SW and butenolide alone. The authors explained these differences by stating that butenolide is a single chemical, while SW contains many various compounds. Soós et al. [16] found that gene expression and protein ubiquitination patterns in maize kernels treated with SW or KAR1 were different. Jain and van Staden [17] proved that SW compounds not only broke seed dormancy but also initiated seedling growth under unfavourable environmental conditions and demonstrated a potential for conditioning of crop seeds.

The aim of the present study was to investigate low-temperature germination of sixty eight *Lupinus angustifolius* lines/cultivars originating from various European regions and to find physiological or biochemical factors affecting the ability of the studied plants to germinate under cold conditions. Moreover, we hypothesized that SW or pre-sowing 3-h hydropriming at room temperature may improve germination of non-dormant seeds of narrow-leaf lupine at 7 °C (cold). Twenty genotypes (the most differentiated in terms of their ability to germinate under cold) were selected from among the tested lines/cultivars. They were analyzed for the effects of seed treatments with SW or 3-h hydropriming on cell membrane permeability, determined by electrolyte leakage as well as dehydrogenase (DA) and amylolytic (A) activity. An additional objective was to elucidate the underlying hormonal changes (ABA and GAs) observed in response to applied seed stimulation treatments.

## 2. Results

### 2.1. Seed Germination Vigour

Statistically significant differences in seed germination vigour (V) were observed among the studied lines/cultivars. Genotype, temperature and treatment were of significance as well. The seeds of most lines/cultivars germinated at a slower rate at 7 °C than at 13 °C. The lines/cultivars differed significantly in their ability to germinate at low temperature and in their response to stimulatory treatments. Table 1 presents V values of 20 lines/cultivars exposed to SW or 3-h hydropriming at the applied temperatures. The main criterion for selection of lines/cultivars was the germination vigour and the percentage of non-germinated seeds in the cold. The chosen lines/cultivars were the most diverse in terms of their ability to germinate under cold.

At 7 °C, control V values ranged between 0.17 (Sethes Fruehe Rote) and 1.53 (Vitabor), while at 13 °C they varied between 0.13 (Brianskij) and 3.34 (No-730). The applied treatments had a stimulating effect on V at both temperatures but the effect was more frequently observed (in 15 out of 20 lines/cultivars) under cold than at 13 °C. Moreover, it was observed mainly in the case of lines/cultivars for which V values at 7 °C were lower than 1.00. The effect of seed treatments was generally stimulating, as there was an improvement observed in 57/80 cases. Hydropriming for 3 h generally increased seed germination vigour, with this effect being noticed in more than 50% of lines/cultivars at both temperatures. The strongest response to this seed pre-treatment was observed in Karo seeds germinating at 7 °C and Kalif seeds germinating at 13 °C, in which 3-h hydropriming enhanced V by 2.4 and 2.7 times, respectively. In most cases smoke water was less effective than 3-h hydropriming. Smoke water increased V values in 28% of cases at both applied temperatures. The highest increase in the presence of SW was observed in Brainskij seeds at 7 °C (2.2 times) and 13 °C (4.8 times). Moreover, the SW-stimulating effect was observed in Sethes Fruehe Rote at 7 °C (1.4 times) and at 13 °C (1.7 times), in Kalif at 7 °C (1.6 times) and at 13 °C (1.3 times), in Karo at 7 °C (1.5 times) and at 13 °C (1.3 times), in Determinant-4 only at 7°C (1.5-fold) and in Kurant only at 13 °C (1.4 times). 

The percentage of non-germinating seeds under cold conditions, presented in Table 2, was calculated six days after sowing.

The most cold-sensitive lines/cultivars were Brianskij (41.2% of non-germinating seeds at 7 °C) and Sethes Fruehe Rote (40.6%), while the most cold-tolerant ones were Kurant (1.5%), No-444 (2.9 %), Determinant-4 (3.0%), and line 95925 (3.2%). Determinant-4 showed the highest number of germinated seeds at both temperatures under both treatments. 

It should be noted that the percentage of non-germinated seeds did not correlate with seed germination vigour (V). Some lines/cultivars needed more time to germinate at unfavourable temperature (with low germination vigour), but demonstrated high final number of germinated seeds, for example Karo and R-7009 × Chittick. Furthermore, Vitabor showed the highest value of V and relatively high number of non-germinated seeds under cold, while germination vigour of No-444 and Determinant-4 was low and the final number of germinated seeds was high. Seeds of Brianskij and Sethes Fruehe Rote germinated very slowly and showed the highest number of non-germinated seeds after six days. 

In many cases, the applied stimulants decreased the number of non-germinating seeds, which was the most visible following exposure to 3-h hydropriming. An improvement in total number of germinating seeds after stimulant application was observed in Brianskij and Sethes Fruehe Rote, the most cold-sensitive cultivars, where hydropriming decreased the percentage of non-germinating seeds at 7 °C by 1.5 and 1.3 times, respectively, and at 13 °C by 12 and 7.4 times, respectively. In more cold-tolerant lines/cultivars, this effect was much weaker, and they germinated much better also without any stimulating treatment.

Smoke water improved germination most effectively at 7 °C in the case of such lines/cultivars as Determinant-4, Kalif, Lazur, Vitabor and No-730. Regarding the final number of germinating seeds under cold, Kazan, R-7009 × Chittick, St. Treb.-4 and Kurant did not respond to any treatment. In some cases, for example for Kazan and St. Treb.-4, even lower germination rates were observed. 

**Figure 1:**
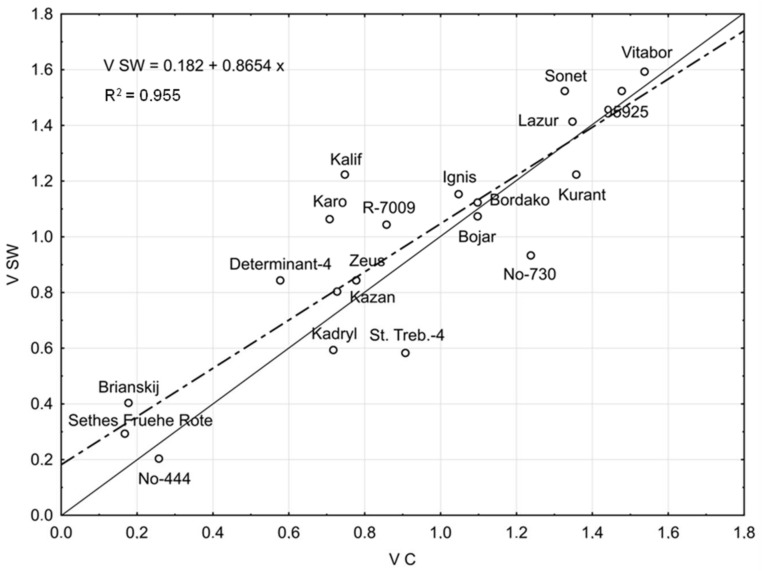
Seed germination vigour (V) of *Lupinus angustifolius* lines/cultivars at 7 °C under smoke water (SW) treatment (V SW) vs. control (V C). Dashed line indicates the regression line. Solid line separates lines/cultivars according to their response to SW: points lying above and below the line correspond to more- and less-responsive line/cultivars, respectively.

**Figure 2:**
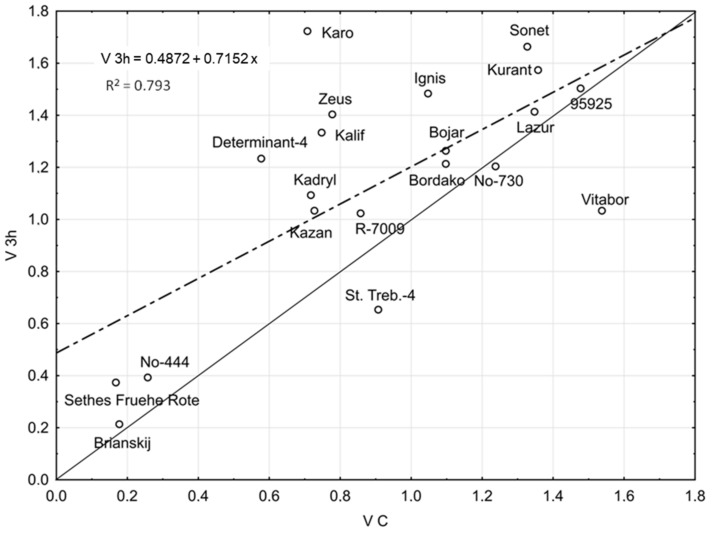
Seed germination vigour (V) of *Lupinus angustifolius* lines/cultivars at 7 °C after 3-h hydropriming (V 3 h) vs. control (V C). Dashed line indicates the regression line. Solid line separates lines/cultivars according to their response to 3-h hydropriming: points lying above and below the line correspond to more- and less-responsive line/cultivars, respectively.

The influence of SW and 3-h hydropriming on seed germination vigour (V) of the studied lines/cultivars at 7 °C relative to control values is illustrated in Figure 1 and Figure 2, respectively. Regression lines were shown along with corresponding equations and baselines dividing lines/cultivars into those more or less responsive to the stimulants. 

As demonstrated in Figure 1, SW enhanced seed germination vigour (V) of Brianskij, Sethes Fruehe Rote, and Determinant-4 (cold sensitive cultivars), Ignis, Kalif, and R-7009 × Chittick (cultivars well germinating under cold), and Lazur, Sonet and Vitabor (cultivars very well germinating under cold). Line/cultivars located below the regression line are those less-responsive to SW treatment.

Three-hour hydropriming improved V values of most studied lines/cultivars, with Karo and Determinant-4 identified as the most responsive to this treatment (Figure 2). The weakest response to all treatments was observed in St. Treb.-4 and Vitabor. 

The results of our analyses demonstrated that germination vigour at cold temperature and responses of studied lines/cultivars to applied treatments stimulating seed germination focused on groups rather than individual lines and cultivars. For this reason, the studied lines/cultivars were divided into three groups according to their control V values at 7 °C: (I) weakly with V range of 0.17–0.58, (II) well with V range of 0.71–1.05, and (III) very well with V range of 1.33–1.48 germinating at cold. Brianskij, Sethes Fruehe Rote, No-444 and Determinant-4 constituted group I. Group II comprised Karo, Kalif, Ignis and Zeus, while group III included Sonet, Lazur, Kurant and line 95925. 

The results presented below are means of particular lines/cultivars included in each group.

### 2.2. Ion Leakage (EL) and Dehydrogenase and Amylolytic Activity

**Figure 3:**
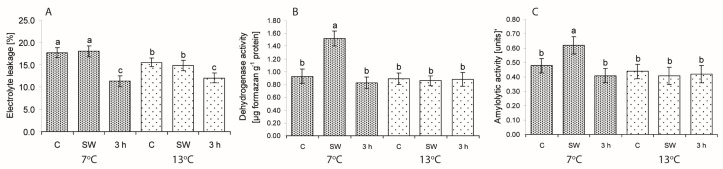
Effect of low (7 °C) and control (13 °C) temperatures and treatments on the percentage of ion leakage (**A**), dehydrogenase (**B**) and amylolytic activity (**C**) in the seeds of lines/cultivars included in Group I (weakly germinating). C—control, non-stimulated seeds; SW—smoke water; 3 h—3-h hydropriming. Values represent means ± SE of four lines/cultivars based on five replicates in the case of DA and A, and on 10 replicates in the case of EL for each line/cultivar. Different superscript letters (a, b, c, ...) in the columns indicate significant differences between means (Duncan’s multiple range test; *p* < 0.05). One unit of amylolytic activity means a decrease in absorbance by 0.02 which released 0.1 mg of starch hydrolyzed in 10 min per 1 mg of protein.

The impact of seed treatments on ion leakage from cells, which is commonly used as an indicator of the degree of cell membrane permeability, as well as dehydrogenase and amylolytic activities in seeds of weakly germinating lines/cultivars are presented in Figure 3.

Three-hour hydropriming reduced ion leakage (EL) at both temperatures (Figure 3A). Activity of dehydrogenases and amylases in the weakly germinating lines were enhanced only by SW at 7 °C (Figure 3B,C). At this temperature, seed vigour (V) correlated with EL (*r* = −0.52; *p* < 0.05) and DA (*r* = 0.35; *p* < 0.05). At 13 °C, V correlated only with amylolytic activity (A) (*r* = 0.42; *p* < 0.05). Correlation between V and DA was weak but significant. 

**Figure 4:**
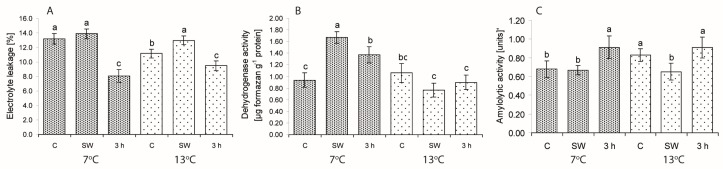
Effect of low (7 °C) and control (13 °C) temperatures and treatments on the percentage of ion leakage (**A**), dehydrogenase (**B**) and amylolytic activity (**C**) in the seeds of lines/cultivars included in Group II (well germinating). C—control, non-stimulated seeds; SW—smoke water; 3 h—3-h hydropriming. Values represent means (±SE) of four lines/cultivars based on five replicates in the case of DA and A, and on 10 replicates in the case of EL for each line/cultivar. Different superscript letters (a, b, c, ...) in the columns indicate significant differences between means (Duncan’s multiple range test; *p* < 0.05). One unit of amylolytic activity means a decrease in absorbance by 0.02, which released 0.1 mg of starch hydrolyzed in 10 min per 1 mg of protein.

In the seeds of well germinating lines/cultivars, cell membrane permeability was reduced also by 3-h hydropriming at both temperatures (Figure 4). Dehydrogenase activity (DA) at 7 °C increased after the treatment with SW. Seed 3-h hydropriming enhanced the activity of DA and A at low temperatures. In this group of lines/cultivars at 7 °C a correlation between seed vigour (V) and cell membrane permeability (EL) (*r* = −0.69; *p* < 0.05) and between V and amylolytic activity (A) (*r* = 0.56; *p* < 0.05) was found. No significant correlation between seed germination vigour (V) and the studied parameters was observed in the seeds germinating at 13 °C. 

**Figure 5:**
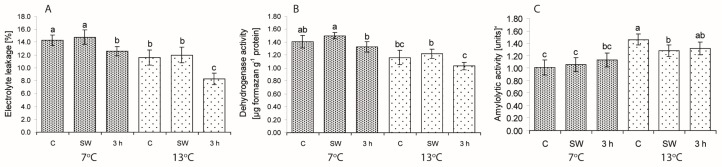
Effect of low (7 °C) and control (13 °C) temperatures and treatments on the percentage of ion leakage (**A**), dehydrogenase (**B**) and amylolytic activity (**C**) in the seeds of lines/cultivars included in Group III (very well germinating). C—control, non-stimulated seeds; SW—smoke water; 3 h—3-h hydropriming. Values represent means (±SE) of four lines/cultivars based on five replicates in the case of DA and A, and on 10 replicates in the case of EL for each line/cultivar. Different superscript letters (a, b, c, ...) in the columns indicate significant differences between means (Duncan’s multiple range test; *p* < 0.05). One unit of amylolytic activity means a decrease in absorbance by 0.02, which released 0.1 mg of starch hydrolyzed in 10 min per 1 mg of protein.

The third group of very well germinating lines/cultivars responded weakly to the seed treatments (Figure 5).

Ion leakage from the seeds germinating at 13 °C under various treatments was smaller than that at 7 °C; but in most cases these differences were not significant. Considerably reduced EL was noticed in the seeds after 3 h treatment at both temperatures compared to that of the control. Dehydrogenase activity (DA) was lower at 13 °C than at 7 °C. Insignificant increase in DA was observed at both temperatures under the influence of SW, while the lowest activity was recorded at 13°C after 3-h hydropriming. Amylolytic activity (A) was higher at 13 °C than at 7 °C. At 7 °C, seed vigour (V) of lines/cultivars belonging to group III correlated only with A (*r* = 0.68; *p* < 0.05).

### 2.3. Hormone Content

In the analysed seeds active gibberellins (GAs), such as GA_1_, GA_3_, GA_4_, GA_5_, GA_6_, and non-active GA_8_ (a product of gibberellin deactivation) were estimated. The group of weakly germinating lines/cultivars showed the lowest level of GA_8_ as compared with group II and III (Table 3). In the lines/cultivars weakly and very well germinating at cold, the level of active gibberellins in control seeds at 7 °C was higher than at 13 °C. In weakly germinating seeds 3-h hydropriming increased the content of gibberellins at 7 °C, while in the seeds of very well germinating lines/cultivars this effect was observed at 13 °C. Smoke water triggered gibberellin accumulation only at 7 °C in the group of well germinating seeds. 

In the imbibed seeds of all groups GA_1_ occurred at the highest level among all detected active gibberellins at both temperatures (Table 4). At 7 °C groups I and II showed greater GA_1_ amount than group III. The weakest germinating group showed greater amount of GA_4_ compared to well and very well germinating groups. In the seeds of groups I and II, GA_5_ occurred at the lowest level. The very well germinating seeds differed from the other seeds with a higher content of GA_6_. Smoke water and 3-h hydropriming nonspecifically affected the content of active GAs in all studied seeds. The GAs content did not depend on germination temperatures. No correlation between GA_8_ or active GAs amount and seed germination vigour (V) was found in any group. However, a correlation was determined between GA_8_ and GA_1_, GA_6_ and GA_4_: *r* = 0.616, *r* = 0.600, and *r* = −0.505 (*p* < 0.05), respectively. 

Seed content of active ABA (free ABA) and non-active form conjugated with glucose (ABA-glc) was quantified. Analyses of ABA level showed a more important role of this hormone than of gibberellins in differentiation of the studied lines/cultivars in terms of their ability to germinate not only at 7 °C but also at 13 °C (Table 5). In the group of lines/cultivars weakly germinating at 7 °C, ABA-glc level was the lowest in the control seeds, while free ABA was the most abundant as compared with the other groups. The lowest level of free ABA characterized the very well germinating seeds. In all groups the content of ABA-glc was significantly higher than of free ABA, especially in both groups of better germinating seeds. Temperature did not influence free ABA content in the control germinating seeds of all groups, while the amount of ABA-glc was higher at 7 °C than at 13 °C. Both treatments increased ABA-glc amount in the seeds of group I at 7 °C and reduced its content in the other groups at both temperatures. Smoke water reduced free ABA amount in the seeds of group I at both temperatures, while in the groups II and III only at 13 °C. Contrary to that, pre-sowing hydropriming reduced free ABA content in all groups at both studied temperatures. A correlation (r = −0.534 at *p* < 0.05) was found only between free ABA level and seed germination vigour (V). In general, an increase in GAs content in the seeds exposed to SW or hydropriming did not correlate with a decrease in ABA level. Surprisingly, the ratio between active gibberellins and free ABA was insignificant for all seed groups, so the data are not presented. No correlation between this ratio and germination vigour was found.

## 3. Discussion

Germinating seeds and seedlings are exposed at critical stages of plant development to various abiotic stresses that pose serious risks to the plants growing near the soil surface. The stresses include drought, salinity and cold [18]. Seeds reaching maturity in a highly dehydrated state exhibit dramatic changes in metabolism [19]. Upon imbibition, dry seeds rapidly consume oxygen, an element required for oxidative phosphorylation that provides energy for intensified metabolic processes. Oxidative phosphorylation and hydrolysis of storage compounds generate reactive oxygen species (ROS), which, if insufficiently scavenged, cause extensive structural and functional damage in cells [20]. Exposure of sensitive seedlings to chilling reduces the number of germinated seeds, decreases root and shoot growth and increases leakage of solutions of low molecular weight metabolites from cells due to a loss of membrane integrity [21]. Cold also plays a key role in breaking seed dormancy, as it may induce GA biosynthesis during early phases of germination [22]. Each plant species has a specific optimal temperature range for germination. Cold may also induce synthesis of cold-shock domain proteins or transcription factors affecting seed germination [23,24]. Narrow-leaf lupine is not a cold-sensitive species and it is sown in early spring. Our study demonstrates that low temperature (7 °C) significantly extends the time necessary for germination. As per our experience (data not shown), the temperature below 7 °C limits narrow-leaf lupine seed germination in the dishes, while 13 °C is optimal for seed germination and growth at early developmental stage of this plant species. In the present study, we found significant differences in low-temperature germination ability among the lines/cultivars of narrow-leaf lupine. Similarly, Nordborg and Bergelson [25] observed considerable differences in low-temperature germination times of 35 ecotypes of *Arabidopsis thaliana*. 

In our investigation most narrow-leaf lupine lines/cultivars did not germinate at 7 °C, even six days after sowing. In the weakly germinating group, on average 2.5 times fewer seeds germinated at the lower than at the higher temperature. In the groups well (II) and very well (III) germinating under cold, this ratio for the seeds germinating at 13 °C and 7 °C was much lower. At this stage of the research, it is difficult to pinpoint the reason for variable germination ability at low temperatures. Genetic background seems to be the major factor controlling germination in a specific climate. We have not found any correlations between the origin of the studied lines/cultivars and their ability to germinate at low temperature. Cultivars from the same country demonstrated both weak and strong germination ability at 7 °C. The seeds of all three groups showed greater efflux of ions from cells under cold as compared with 13 °C. Kaur et al. [26] noticed a decrease in the germination vigour of *Cicer arietinum* seeds accompanied by greater EL and a decrease in seed dehydrogenase activity in cold conditions. In our experiment, the seed germination vigour of weakly and well germinating groups (I and II) depended on EL, which may indicate a degree of plasma membrane permeability. The weakly germinating lines/cultivars experienced the greatest ion leakage from seed cells at low temperature, while the group of very well germinating lines/cultivars (group III) showed the lowest ion efflux; this feature thus seems to be a major determinant of low-temperature germination ability. Cell membrane conditions affect all metabolic processes, and, especially in germinating seeds, oxidative phosphorylation. According to Leopold and Musgrave [27], a decrease in TTC reduction suggested a loss of mitochondrial stability. The relationship between germination vigour at low temperature and dehydrogenase activity may be explained by the necessity of providing energy for de novo synthesis of many compounds and hydrolysis of storage compounds in the endosperm. In our experiment, seed germination vigour correlated with dehydrogenase activity only in the weakly germinating cultivars. In addition, the seeds in group I had lower amylolytic activity than those in groups II and III. Weak germination observed under cold conditions seemed to depend on such factors as cell membrane permeability, activity of dehydrogenases and amylolytic activity. In contrast, the vigour of very well germinating seeds depended only on amylolytic activity. 

One of the aims of our research was to improve seed germination of narrow-leaf lupine at low temperature via application of smoke water as well as pre-sowing 3-h hydropriming at 20 °C. The results indicate that the hydropriming significantly increased germination vigour of most studied lines/cultivars. Water uptake at room temperature was rapid during the first three hours, and subsequent transfer of seeds to low temperature did not cause any major damage to the structural membranes. It is difficult to tell whether in such a short time any rearrangement of cytoplasmic membranes occurred, which in nature is a typical physiological response of plants to changes in the temperature and degree of the cytoplasm hydration. This question requires molecular analysis of the changes in cell membrane structure taking place during the initial three hours of imbibition. According to Bewley [2] membranes return to a more stable configuration shortly after rehydration. Based on our observations, we conclude that the initial hydration of seeds protected their cell membranes against cold-induced damage as manifested by a decrease in ion leakage from seed cells. Similar results were obtained by Dubert and Filek [5], who reported that 100% of hydroprimed soybean seeds germinated at 5 °C without showing any structural or functional disorders. They postulated that seed imbibition at low temperature requires a quick reconstruction of the cell membrane of dry seeds into a new structure characteristic of germinating seeds. This reconstruction requires an activation of appropriate enzymatic processes that run too slowly at low temperature, which in turn causes damage to the cell membranes. Contrary to that, hydropriming at room temperature allows for harmonizing the swelling of cells with the processes of remodeling of their membranes. Smoke water may mobilise seed metabolism via activation of dehydrogenases engaged in glycolysis and the Krebs cycle. Karrikins contained in SW were confirmed to enhance germination of approximately 1200 plant species worldwide [15]. However, it should be noticed that most studies on the stimulating action of SW on seed germination focused on dormancy break. Although the molecular mechanism underlying this response remains unknown, some investigations demonstrated a key role of GAs and NO_2_ in smoke-induced seed germination [12,28]. Seeds of many plant species originating from various world regions show a positive response to the compounds of smoke. Apart from typical post-fire flora, responsive species include crop plants such as *Lactuca sativa*, *Zea mays*, *Apium graveolens* and *Avena fatua* [29]. 

Most studies investigating the role of antagonistic hormones, such as GAs and ABA, were performed on dormant seeds [30]. One of the aims of our research was to find out whether the content of ABA and GAs determine the germination ability of non-dormant seeds of narrow-leaf lupine under cold. Both hormones occur in the seeds in active and non-active forms, for example conjugated in the case of ABA, or hydroxylated in the case of GA_1_ to GA_8_. According to Halińska and Lewak [31], the equivalence between changes in active and non-active GAs suggests that the latter are also involved in the control of physiological levels of active gibberellins. In our study, we determined the levels of both forms of ABA and GAs, and the results indicated that only active forms of ABA determined the germination ability of non-dormant seeds of narrow-leaf lupine. In our experiment, such active forms as GA_1_, GA_3_, GA_4_, GA_5_, GA_6_ as well as non-active GA_8_ were quantified. The most common active gibberellin forms are GA_1_, GA_3_ and GA_4_. Universal occurrence of GA_1_ and GA_4_ in plants suggests that they are functionally active forms and co-occur with their biosynthetic precursors and metabolites, which are often present at much higher concentrations than the hormones themselves [32,33]. Our analysis showed that the seeds of narrow-leaf lupine contained the largest amounts of GA_8_ as compared with individual free gibberellins. We found differences in gibberellin accumulation between lines/cultivars varying in terms of germination ability at cold. This was particularly visible for GA_8_ accumulation pattern. Generally, the groups of well and very well germinating seeds showed higher GA_8_ levels than the group of weakly germinating ones. According to Ross et al. [34], seeds approaching maturity often contain high levels of inactive GA, ensuring against concentrations of bioactive GAs in mature seed that could provoke premature germination and abnormal seedling growth. It seems that the capability of maintaining high rate of GA metabolism is more important for germination efficiency than total GAs accumulation. Our opinion could be confirmed by positive correlation between GA_1_ and GA_8_ as well as negative correlation between GA_4_ and GA_8_ content. Gibberellin GA_8_ is a direct metabolite of GA_1_ deactivation, while GA_4_ is a part of metabolic pathway parallel to GA_1_ [33,35]. On the other hand, GA_4_ bioactivity is greater than GA_1_ [36,37], thus, they mutually affect their accumulation as well as metabolism rate. Analysing the role of individual gibberellins in germination only on the basis on their accumulation in seeds is not easy due to their very complicated metabolic pathway providing many possibilities of synthesis and degradation as well as conversion of one compound into another. For example GA_5_ may be converted into GA_6_ or GA_3_ [33]. Statistical analysis did not reveal any correlation between either GA_8_ or total GA level in the seeds and their germination vigour at both studied temperatures. Seed germination seemed to be affected rather by reciprocal equilibrium of the gibberellins than by the content of individual GAs alone. Chien et al. [37] reported that the combination of GA_4_ and GA_7_ had a greater effect on the germination of dormant *Taxus mairei* seeds than GA_4_ alone. A prevailing view is that seed germination is controlled by ABA and GAs ratio [30]. In our study, the ratio of active forms of gibberellins to free abscisic acid had no effect on the germination ability. Similarly to GAs, only the levels of free ABA correlated with lupine seed germination vigour. The stimulating effect of 3-h hydropriming on germination vigour was caused not only by a decrease of cell membrane permeability but also by a reduction in ABA content in the imbibed seeds. Smoke water and hydropriming were more effective in ABA reduction than in GAs increase. Contrary to our results, Chiwocha et al. [38] reported that some studies on dormant *Arabidopsis* seeds demonstrated no effect of KAR_1_ on endogenous ABA and GAs content. It seems that this effect is species specific. It is worth mentioning that SW contains many various compounds exerting positive and negative impact on seed germination, so the effect of a single compound could not be so extensive compared to SW [16]. Also our other research on the effects of butenolide, i.e., 2(5*H*)-furanone on lupine seed germination (not yet published) demonstrated its less intensive impact than SW. Although the level of ABA seems to play a key role in seed germination, a decrease in the endogenous ABA does not always correlate with germination [39] or is not sufficient to break dormancy in some dormant seeds [40]. Humplik et al. [41] showed that ABA was essential for hypocotyl elongation and that appropriate control of the endogenous level of ABA was required in order to drive the growth of etiolated seedlings.

Taking into account positive effects of SW and hydropriming on seed germination at low temperature, we hope that they could be used also in the field cultivation of crop plants. Both pre-sowing treatments do not change surface properties of seeds which can still be sown using standard seed drills. 

## 4. Materials and Methods

### 4.1. Plant Material

Seeds of 20 lines/cultivars of *Lupinus angustifolius* were obtained from the collections of the Smolice Plant Breeding Station, Przebędowo, IHAR group, Poland, and the Poznań Plant Breeding Station, Wiatrowo, Poland. The seeds were collected in autumn 2015, and the experiments were performed in March 2016 in laboratory conditions. The seeds were surface sterilised in 70% ethanol and washed three times with sterile water. Plastic Petri dishes (9 cm) containing filter paper were filled with 15 cm^3^ of one of the following solutions: distilled water (control and 3-h hydroprimed seeds) or a commercial condensate of SW (DESPOL, ENDERS group, Poland) diluted 1:1000 (*v*/*v*) with distilled water. The applied dilution and duration of hydropriming were chosen based on a preliminary study. Fifteen seeds of each line/cultivar were placed in a dish for each treatment. Each dish constituted one replicate, with five replicates performed per treatment (five dishes for each treatment and temperature per line/cultivar). The seeds germinated in darkness in phytotron chambers at 7 °C (cold) or 13 °C (control). Hydroprimed seeds were soaked in dishes with distilled water at 20 °C for 3 h before their transfer to the final temperature of 7 °C or 13 °C. As per our experience, the temperature below 7 °C limits narrow-leaf lupine seed germination in the dishes, while 13 °C is optimal temperature for seed germination and growth at early developmental stage of this plant species.

### 4.2. Determination of Seed Germination Vigour (V)

Germination vigour for each dish, estimated four days after sowing, was calculated based on hypocotyl length using the following visual scale: 0—no germination; 1—hypocotyl length of 1 mm; 2—hypocotyl length of 2–3 mm; 3—hypocotyl length of 4–7 mm; and 4—hypocotyl length greater than 7 mm. The V value was calculated for each replicate (dish) according to the Equation (1):

V = [*n*_0_ × 0 + ... + *n*_4_ × 4]/*N* (1)

where *n*_x_ is the number of seeds corresponding to a given hypocotyl length and *N* is total number of seeds in a dish. 

Six days after sowing, the final number of non-germinated seeds was counted and expressed as the percentage of all sown seeds in each treatment and temperature. V values estimated at low temperature were used to identify the lines/cultivars responding in the strongest and weakest manner to: (1) the cold, in terms of an ability to germinate at low temperature; (2) the stimulatory treatments. Seeds of the twenty selected lines/cultivars were sown again into Petri dishes and treated with SW or 3-h hydropriming as described above. Next, they were subjected to analyses performed in the seeds collected after 24 h of the imbibition. 

### 4.3. Electrolyte Leakage (EL)

Seeds collected from each line/cultivar and a combination of temperature/treatment were placed into vials containing 13 cm^3^ of ultrapure water (one seed per vial), and shaken (100 rpm) at 20 °C. After 24 h, electrical conductivity (E1) was measured using a conductometer (CI 317, Elmetron, Poland). The vials with samples were frozen for 24 h at −80 °C, then they were thawed and shaken again. Conductivity measurements were repeated and the obtained values represented total ion content (E2) of the seed. Membrane permeability was expressed as the percentage of total EL according to the Equation (2):

EL = [E1 × 100]/E2(2)

All measurements were performed for 10 biological replicates.

### 4.4. Dehydrogenase Activity Assay

The activity of dehydrogenase pool (DA) in seeds was measured according to Steponkus and Lanphear [42] with a slight modification. Seeds collected from each line/cultivar and the combination of temperature/treatment were weighed and placed into plastic vials along with 3 cm^3^ of a reaction mixture containing 1.5 cm^3^ of 0.4% (*v/w*) aqueous 2,3,5-triphenyltetrazolium chloride (TTC) and 1.5 cm^3^ of 0.1 M phosphate buffer (pH 7.5). The seeds were incubated for 3 h at 37 °C in the dark. Then they were homogenised with 5 cm^3^ of 96% ethanol to extract triphenylformazan, a product of TTC reduction by seed dehydrogenases. The extract was centrifuged at 16,000× *g* for 5 min. Finally, absorbance of the supernatant was measured at 485 nm using an Ultrospec 2100 Pro spectrophotometer (Amersham Biosciences, Little Chalfont, UK). DA activity was expressed as μg of formazan per 1 g of protein on the basis of the trend line. Protein concentrations in seeds were determined spectrophotometrically according to Bradford [43] at 595 nm using the Bio-Rad (Munich, Germany) protein assay with bovine serum albumin as a standard. Analyses of DA activity as well as protein amount were performed for five biological replicates.

### 4.5. Amylolytic Activity (A) Assay

The amylolytic activity in the seeds was measured according to Heinkel [44]. Seeds collected from each line/cultivar and the combination of temperature/treatment were weighed and homogenised in 5 cm^3^ of 0.1 M phosphate buffer (pH 7.0), and then centrifuged at 16,000× *g* for 5 min. One cm^3^ of the supernatant was shaken with 100 μL of 2% Lugol’s reagent and 200 μL of 0.5% starch solution, and initial absorbance was immediately read at 595 nm using an Ultrospec 2100 Pro spectrophotometer (Amersham Biosciences, Little Chalfont, UK). The second absorbance measurement was carried out after 10 min. Amylolytic activity was expressed as units, where one unit means a decrease in absorbance by 0.02, which released 0.1 mg of starch hydrolyzed in 10 min per 1 mg of protein. Analyses were conducted for five biological replicates.

### 4.6. Analyses of GA and ABA Content

Phytohormones were measured according to Hura et al. [45]. Seeds from each line/cultivar and the combination of temperature/treatment were freeze-dried and pulverised (MM400, Retch, Germany). The samples were spiked with stable isotope labelled internal standards (ISTD, [2H2]gibberellin A_1_, [2H2]gibberellin A_4_ and [2H6]*cis*,*trans*-abscisic acid) and extracted. The extracts were cleaned up on SPE cartridges (BondElutPlexa PCX, 30 mg, 1 mm, Agilent, Santa Clara, CA, USA), and used for ultra-high-performance liquid chromatography (UHPLC) analyses. The system consisted of UHPLC (Agilent Infinity 1260, Agilent, Waldbronn, Germany) and a triple quadruple mass spectrometer (Agilent 6410, Agilent, Santa Clara, CA, USA) with electro-spray ionization (ESI). Detailed measurement conditions were given by Dziurka et al. [46]. Phytohormones were quantitatively assessed based on calibration curves acquired for pure standards taking account ISTD recoveries: [2H2]gibberellin A_1_ for GA_8_, GA_1_, GA_3_, GA_6_; [2H2]gibberellin A_4_ for GA_5_ and GA_4_, and [2H6]*cis*,*trans*-abscisic acid for all ABA species. All the standards were from OlChemIm (Olomouc, Czech Republic) of the highest available purity. Analyses were performed in three biological replicates. The data are presented as fmol g^−1^ DW (femtomol g^−1^ dry weight).

### 4.7. Statistical Analyses

The obtained data were analyzed using a two-way ANOVA (STATISTICA 12 software, StatSoft, Tulsa, OK, USA). The percentage data before analysis were transformed according to the Equation (3):

(3) $$y=arcsin\sqrt{x}$$

Means and standard errors were calculated. Comparisons of the treatments were carried out according to Duncan’s multiple range test at *p* < 0.05. Significance of linear correlation (Pearson’s coefficient) was calculated at *p* < 0.05. 

## 5. Conclusions

Seed germination vigour of all studied narrow-leaf lupine lines/cultivars under low temperature conditions depends mainly on the genotype, degree of cell membrane damage, amylolytic activity, and ABA content. In the case of lines/cultivars weakly germinating at low temperature, seed germination vigour depends additionally on dehydrogenase activity. Stimulating effect of smoke water and seed hydropriming on seed germination vigour is most visible in the case of cold sensitive genotypes as compared with cold tolerant ones. 

Three-hour hydropriming protects seeds of most studied narrow-leaf lupine lines/cultivars against cold-induced damage during germination. This protective effect is mainly due to decreased cell membrane permeability and ABA level. Smoke water may stimulate germination of narrow-leaf lupine seeds via activation of the dehydrogenases engaged in the respiration process. Smoke water reduced ABA content mainly in the seeds of lines/cultivars weakly germinating under cold, while hydropriming decreased ABA level in the seeds of all studied lines/cultivars at applied temperatures. 

## Figures and Tables

**Table 1:** Effects of smoke water (SW) and 3-h hydropriming (3 h) on seed germination vigour (V) at cold (7 °C) and control (13 °C) temperatures in the investigated *Lupinus angustifolius* lines/cultivars. Control—non-stimulated seeds.

Line/Cultivar and Origin	Temperature [°C]	V		
		Control	SW	3 h
Brianskij (Russia)	7	0.18 ± 0.03 ^c^	0.40 ± 0.06 ^a^	0.22 ± 0.03 ^b^
	13	0.13 ± 0.02 ^c^	0.63 ± 0.09 ^a^	0.21 ± 0.03 ^b^
Sethes Fruehe Rote (Germany)	7	0.17 ± 0.03 ^c^	0.29 ± 0.04 ^b^	0.37 ± 0.04 ^a^
	13	1.01 ± 0.15 ^b^	1.44 ± 0.22 ^a^	1.06 ± 0.16 ^b^
No-444 (Russia)	7	0.26 ± 0.04 ^b^	0.20 ± 0.03 ^b^	0.39 ± 0.06 ^a^
	13	0.40 ± 0.05 ^a^	0.44 ± 0.06 ^a^	0.26 ± 0.04 ^b^
Determinant-4 (Belarus)	7	0.58 ± 0.07 ^c^	0.84 ± 0.09 ^b^	1.23 ± 0.12 ^a^
	13	0.60 ± 0.07 ^b^	0.64 ± 0.07 ^b^	1.06 ± 0.13 ^a^
Kalif (Poland)	7	0.75 ± 0.11 ^b^	1.22 ± 0.18 ^a^	1.33 ± 0.16 ^a^
	13	0.72 ± 0.11 ^c^	0.94 ± 0.14 ^b^	1.92 ± 0.29 ^a^
Kadryl (Poland)	7	0.72 ± 0.11 ^b^	0.59 ± 0.09 ^c^	1.09 ± 0.12 ^a^
	13	0.78 ± 0.12 ^c^	1.04 ± 0.15 ^b^	1.51 ± 0.23 ^a^
Kazan (Poland)	7	0.73 ± 0.09 ^b^	0.85 ± 0.12 ^b^	1.03 ± 0.15 ^a^
	13	0.88 ± 0.13 ^b^	0.89 ± 0.13 ^b^	1.52 ± 0.19 ^a^
Zeus (Poland)	7	0.78 ± 0.12 ^b^	0.84 ± 0.11 ^b^	1.40 ± 0.22 ^a^
	13	0.96 ± 0.13 ^b^	0.99 ± 0.13 ^b^	1.78 ± 0.24 ^a^
R-7009 × Chittick (Poland)	7	0.86 ± 0.14 ^b^	1.04 ± 0.16 ^a^	1.02 ± 0.14 ^a^
	13	1.21 ± 0.18 ^a^	1.03 ± 0.15 ^a^	0.85 ± 0.12 ^b^
Karo (Poland)	7	0.71 ± 0.09 ^c^	1.06 ± 0.16 ^b^	1.72 ± 0.24 ^a^
	13	2.45 ± 0.37 ^b^	3.09 ± 0.40 ^a^	2.34 ± 0.35 ^b^
Lazur (Poland)	7	1.35 ± 0.21 ^a^	1.41 ± 0.19 ^a^	1.42 ± 0.21 ^a^
	13	1.47 ± 0.22 ^b^	1.43 ± 0.20 ^b^	1.95 ± 027 ^a^
Ignis (Poland)	7	1.05 ± 0.16 ^b^	1.15 ± 0.17 ^b^	1.48 ± 0.22 ^a^
	13	3.06 ± 0.46 ^a^	3.12 ± 0.47 ^a^	2.87 ± 0.40 ^a^
Vitabor (Germany)	7	1.53 ± 0.23 ^a^	1.59 ± 0.22 ^a^	1.06 ± 0.15 ^b^
	13	2.67 ± 0.37 ^ab^	2.96 ± 0.44 ^a^	2.33 ± 0.33 ^b^
No-730 (Russia)	7	1.24 ± 0.19 ^a^	0.93 ± 0.13 ^a^	1.20 ± 0.18 ^b^
	13	3.34 ± 0.51 ^a^	3.35 ± 0.50 ^a^	3.32 ± 0.49 ^a^
Sonet (Poland)	7	1.33 ± 0.20 ^a^	1.52 ± 0.23 ^a^	1.66 ± 0.23 ^a^
	13	2.53 ± 0.38 ^a^	2.85 ± 0.43 ^a^	3.06 ± 0.45 ^a^
St. Treb.-4 (Germany)	7	0.91 ± 0.14 ^a^	0.58 ± 0.07 ^b^	0.65 ± 0.11 ^b^
	13	2.86 ± 0.43 ^a^	2.61 ± 0.39 ^a^	2.85 ± 0.43 ^a^
95925 (Russia)	7	1.48 ± 0.22 ^a^	1.52 ± 0.21 ^a^	1.50 ± 0.21 ^a^
	13	3.00 ± 0.42 ^a^	2.39 ± 0.26 ^b^	2.56 ± 0.31 ^ab^
Bojar (Poland)	7	1.10 ± 0.15 ^a^	1.07 ± 0.16 ^a^	1.26 ± 0.18 ^a^
	13	0.79 ± 0.12 ^b^	0.80 ± 0.11 ^b^	1.30 ± 0.19 ^a^
Bordako (Germany)	7	1.10 ± 0.17 ^a^	1.12 ± 0.17 ^a^	1.21 ± 0.18 ^a^
	13	3.27 ± 0.50 ^a^	2.80 ± 0.42 ^a^	3.26 ± 0.48 ^a^
Kurant (Poland)	7	1.36 ± 0.16 ^a^	1.22 ± 0.18 ^a^	1.57 ± 0.19 ^a^
	13	1.17 ± 0.18 ^c^	1.62 ± 0.23 ^b^	2.53 ± 0.33 ^a^

Values represent means (*n* = 5) ± SE. Different superscript letters (a, b, c, ...) in the rows for each temperature indicate significant differences between means (Duncan’s multiple range test; *p* < 0.05).

**Table 2:** Percentage of non-germinating seeds in the studied *Lupinus angustifolius* lines/cultivars at cold (7 °C) and control (13 °C) temperatures following treatment with smoke water (SW) or 3-h hydropriming (3 h). Control—non-stimulated seeds.

Line/Cultivar	Temperature [°C]	V		
		Control	SW	3 h
Brianskij	7	41.2 ± 3.7 ^a^	37.1 ± 3.0 ^b^	28.1 ± 2.4 ^c^
	13	30.8 ± 2.7 ^a^	7.5 ± 0.6 ^b^	2.6 ± 0.2 ^c^
Sethes Fruehe Rote	7	40.6 ± 3.6 ^b^	49.2 ± 4.1 ^a^	30.8 ± 3.2 ^c^
	13	22.9 ± 1.8 ^a^	13.6 ± 1.4 ^b^	3.1 ± 0.3 ^c^
No-444	7	2.9 ± 0.3 ^b^	2.9 ± 0.2 ^b^	8.8 ± 0.9 ^a^
	13	0 ^b^	2.6 ± 0.2 ^a^	2.6 ± 0.2 ^a^
Determinant-4	7	3.0 ± 0.3 ^a^	0 ^b^	0 ^b^
	13	0 ^a^	0 ^a^	0 ^a^
Kalif	7	4.4 ± 0.4 ^a^	1.6 ± 0.2 ^b^	1.4 ± 0.1 ^b^
	13	2.9 ± 0.2 ^b^	1.5 ± 0.1 ^c^	4.3 ± 0.4 ^a^
Kadryl	7	13.4 ± 1.2 ^a^	1.8 ± 1.1 ^c^	4.5 ± 0.3 ^b^
	13	6.6 ± 0.7 ^a^	2.7 ± 0.1 ^c^	4.0 ± 0.4 ^b^
Kazan	7	11.1 ± 1.1 ^b^	19.4 ± 1.7 ^a^	12.5 ± 1.3 ^b^
	13	0 ^a^	0 ^a^	0 ^a^
Zeus	7	8.2 ± 0.8 ^a^	6.2 ± 0.7 ^b^	3.1 ± 0.2 ^c^
	13	1.5 ± 0.1 ^b^	4.3 ± 0.4 ^a^	0 ^c^
R-7009 × Chittick	7	2.0 ± 0.1 ^a^	2.0 ± 0.1 ^a^	2.0 ± 0.1 ^a^
	13	3.8 ± 0.3 ^a^	2.9 ± 0.2 ^b^	3.8 ± 0.3 ^a^
Karo	7	1.5 ± 0.1 ^b^	0 ^c^	3.0 ± 0.3 ^a^
	13	7.9 ± 0.8 ^a^	1.6 ± 0.1 ^b^	0 ^c^
Lazur	7	7.7 ± 0.5 ^a^	2.8 ± 0.3 ^b^	0 ^c^
	13	1.5 ± 0.2 ^a^	0 ^b^	0 ^b^
Ignis	7	6.4 ± 0.4 ^a^	4.5 ± 0.3 ^b^	6.2 ± 0.5 ^a^
	13	5.8 ± 0.6 ^a^	4.3 ± 0.5 ^b^	4.2 ± 0.3 ^b^
Vitabor	7	7.9 ± 0.5 ^a^	1.6 ± 0.1 ^b^	0 ^c^
	13	1.6 ± 0.2 ^a^	0 ^b^	0 ^b^
No-730	7	3.2 ± 0.3 ^b^	0 ^c^	4.5 ± 0.4 ^a^
	13	3.2 ± 0.3 ^a^	1.4 ± 0.1 ^b^	1.4 ± 0.1 ^b^
Sonet	7	5.9 ± 0.5 ^a^	4.5 ± 0.5 ^b^	0 ^c^
	13	4.2 ± 0.4 ^a^	4.2 ± 0.3 ^a^	1.4 ± 0.1 ^b^
St. Treb.-4	7	6.0 ± 0.5 ^c^	11.9 ± 1.2 ^a^	7.8 ± 0.8 ^b^
	13	4.3 ± 0.5 ^b^	5.3 ± 0.6 ^a,b^	5.8 ± 0.6 ^a^
95925	7	3.2 ± 0.3 ^a^	1.5 ± 0.1 ^b^	0 ^c^
	13	0 ^a^	0 ^a^	0 ^a^
Bojar	7	10.9 ± 0.8 ^a^	8.6 ± 0.7 ^b^	8.6 ± 0.7 ^b^
	13	5.8 ± 0.6 ^a^	5.9 ± 0.6 ^a^	2.9 ± 0.2 ^b^
Bordako	7	7.7 ± 0.7 ^a^	7.8 ± 0.8 ^a^	4.6 ± 0.3 ^b^
	13	5.8 ± 0.6 ^a^	1.4 ± 0.2 ^b^	0 ^c^
Kurant	7	1.5 ± 0.2 ^b^	1.5 ± 0.1 ^b^	3.1 ± 0.2 ^a^
	13	0 ^b^	1.4 ± 0.1 ^a^	0 ^b^

Values represent means (*n* = 5) ± SE. Different superscript letters (a, b, c, ...) in the rows for each temperature indicate significant differences between means (Duncan’s multiple range test; *p* < 0.05).

**Table 3:** Effect of smoke water (SW) and 3-h hydropriming (3 h) on GA_8_ and active gibberellin content (fmol g^−1^ DW) in the seeds of *Lupinus angustifolius* lines/cultivars germinating at low (7 °C) and control (13 °C) temperature categorized into three groups: I, weakly germinating; II, well germinating; III, very well germinating. Control—non-stimulated seeds.

Temperature [°C]	Treatment	Group I		Group II		Group III	
		GA_8_	GAs	GA_8_	GAs	GA_8_	GAs
7	Control	128 ± 15 ^b^	988 ± 129 ^b^	458 ± 52 ^b^	598 ± 77 ^b^	594 ± 71 ^a^	904 ± 91 ^a^
	SW	74 ± 8 ^c^	666 ± 80 ^d^	601 ± 72 ^a^	810 ± 97 ^a^	392 ± 43 ^c^	675 ± 61 ^c^
	3 h	149 ± 17 ^a^	1183 ± 153 ^a^	433 ± 47 ^b^	449 ± 53 ^c^	427 ± 46 ^b^	783 ± 74 ^b^
13	Control	64 ± 7 ^d^	478 ± 57 ^e^	420 ± 50 ^b^	704 ± 77 ^b^	304 ± 33 ^c^	630 ± 72 ^c^
	SW	115 ± 13 ^b^	750 ± 97 ^c^	384 ± 46 ^c^	644 ± 84 ^b^	326 ± 35 ^c^	642 ± 73 ^c^
	3 h	40 ± 5 ^e^	338 ± 44 ^f^	442 ± 48 ^b^	466 ± 55 ^c^	308 ± 36 ^c^	839 ± 89 ^a^

Values shown for each group are means ± SE of four lines/cultivars based on three replicates for each line/cultivar. Different superscript letters (a, b, c, ...) in the columns indicate significant differences between means (Duncan’s multiple range test; *p* < 0.05). Following active gibberellins (GAs) were determined: GA_1_, GA_3_, GA_4_, GA_5_, GA_6_.

**Table 4:** Effect of smoke water (SW) and 3-h hydropriming (3 h) on active gibberellin content [fmol g^−1^ DW] in the seeds of *Lupinus angustifolius* germinating at low (7 °C) and control (13 °C) temperature. The lines/cultivars were categorized into three groups: I, weakly germinating; II, well germinating; III, very well germinating. Control—non-stimulated seeds.

Gibberellin	Control		Smoke Water		3-h Hydropriming	
	7 °C	13 °C	7 °C	13 °C	7 °C	13 °C
Group I						
GA_1_	450 ± 49 ^a^	167 ± 16 ^d^	210 ± 18 ^c^	205 ± 18 ^c^	367 ± 33 ^b^	108 ± 9 ^e^
GA_3_	229 ± 25 ^b^	138 ± 12 ^d^	165 ± 14 ^c^	239 ± 22 ^b^	413 ± 37 ^a^	59 ± 6 ^e^
GA_4_	183 ± 20 ^c^	120 ± 10 ^e^	184 ± 17 ^c^	214 ± 19 ^b^	262 ± 24 ^a^	140 ± 15 ^d^
GA_5_	33 ± 3 ^a^	18 ± 2 ^b^	6 ± 3 ^b^	17 ± 2 ^b^	13 ± 2 ^c^	13 ± 1 ^c^
GA_6_	93 ± 11 ^b^	5 ± 3 ^d^	92 ± 8 ^b^	52 ± 4 ^c^	128 ± 12 ^a^	8 ± 2 ^e^
Group II						
GA_1_	401 ± 40 ^a^	411 ± 36 ^a^	436 ± 39 ^a^	392 ± 43 ^a^	277 ± 25 ^b^	273 ± 22 ^b^
GA_3_	89 ± 9 ^a^	43 ± 9 ^b^	54 ± 5 ^b^	87 ± 9 ^a^	85 ± 8 ^a^	2 ± 8 ^a^
GA_4_	41 ± 3 ^c^	42 ± 9 ^c^	9 ± 8 ^a^	74 ± 6 ^b^	8 ± 3 ^c^	41 ± 4 ^c^
GA_5_	26 ± 2 ^a^	22 ± 2 ^b^	13 ± 2 ^d^	7 ± 3 ^a^	13 ± 2 ^d^	7 ± 2 ^c^
GA_6_	41 ± 5 ^d^	187 ± 17 ^b^	208 ± 19 ^a^	2 ± 9 ^c^	6 ± 3 ^e^	44 ± 4 ^d^
Group III						
GA_1_	321 ± 28 ^a,b^	267 ± 24 ^b^	295 ± 21 ^b^	294 ± 26 ^b^	366 ± 32 ^a^	281 ± 25 ^b^
GA_3_	236 ± 21 ^a^	94 ± 7 ^d^	186 ± 17 ^b^	135 ± 12 ^c^	198 ± 22 ^b^	158 ± 14 ^c^
GA_4_	63 ± 6 ^c^	7 ± 7 ^b^	4 ± 4 ^c^	60 ± 7 ^c^	98 ± 9 ^b^	220 ± 20 ^a^
GA_5_	87 ± 8 ^a^	61 ± 5 ^b^	45 ± 3 ^c^	3 ± 4 ^c^	32 ± 3 ^d^	21 ± 2 ^e^
GA_6_	197 ± 17 ^a^	111 ± 9 ^c^	96 ± 9 ^c,d^	108 ± 18 ^c^	89 ± 9 ^d^	159 ± 14 ^b^

Values shown for each group are means ± SE of four lines/cultivars based on three replicates for each line/cultivar. Different superscript letters (a, b, c, ...) in the rows indicate significant differences between means (Duncan’s multiple range test; *p* < 0.05).

**Table 5:** Effect of smoke water (SW) and 3-h hydropriming (3 h) on conjugated (ABA-glc) and free abscisic acid (ABA) content [fmol g^−1^ of DW] in the seeds of *Lupinus angustifolius* lines/cultivars germinating at low (7 °C) and control (13 °C) temperature categorized into three groups: I, weakly germinating; II, well germinating; III, very well germinating. Control – non-stimulated seeds. .

Temperature [°C]	Treatment	Group I		Group II		Group III	
		ABA-glc	ABA	ABA-glc	ABA	ABA-glc	ABA
7	Control	573 ± 63 ^c^	595 ± 71 ^a^	1684 ± 202 ^a^	425 ± 51 ^a^	1764 ± 194 ^a^	204 ± 26 ^b^
	SW	632 ± 69 ^b^	184 ± 23 ^d^	1324 ± 145 ^b^	469 ± 62 ^a^	908 ± 100 ^c^	279 ± 39 ^a^
	3 h	842 ± 92 ^a^	306 ± 42 ^b^	1140 ± 125 ^c^	249 ± 29 ^b^	1196 ± 143 ^b^	143 ± 17 ^c^
13	Control	642 ± 70 ^b^	578 ± 10 ^a^	1319 ± 145 ^b^	419 ± 62 ^a^	931 ± 102 ^c^	216 ± 27 ^b^
	SW	652 ± 71 ^b^	242 ± 31 ^c^	1046 ± 104 ^c^	284 ± 36 ^b^	752 ± 90 ^d^	105 ± 12 ^e^
	3 h	628 ± 75 ^b^	178 ± 21 ^d^	708 ± 77 ^d^	276 ± 35 ^b^	754 ± 90 ^d^	126 ± 16 ^d^

Values shown for each group are means ± SE of four lines/cultivars based on three replicates for each line/cultivar. Different superscript letters (a, b, c, ...) in the columns indicate significant differences between means (Duncan’s multiple range test; *p* < 0.05). ABA means total of (+)-*trans*,*trans*-abscisic acid and (±)-*cis*,*trans*-abscisic acid.

## Abbreviations

3 h	seed hydropriming for 3 h at 20 °C
ABA	abscisic acid
A	amylolytic activity
DA	activity of dehydrogenase pool measured by 2,3,5-triphenyltetrazolium chloride reduction
GAs	gibberellins
EL	electrolyte leakage
SW	smoke water
TTC	2,3,5-triphenyltetrazolium chloride
V	seed germination vigour
